# Specimen Shape and Elution Time Affect the Mineralization and Differentiation Potential of Dental Pulp Stem Cells to Biodentine

**DOI:** 10.3390/jfb15010001

**Published:** 2023-12-19

**Authors:** Valene Phang, Ritika Malhotra, Nah Nah Chen, Kyung-San Min, Victoria Soo Hoon Yu, Vinicius Rosa, Nileshkumar Dubey

**Affiliations:** 1 Faculty of Dentistry, National University of Singapore, Singapore 119085, Singapore; valenephang@gmail.com (V.P.); m.ritika@nus.edu.sg (R.M.); victoria@nus.edu.sg (V.S.H.Y.); 2 National Dental Centre Singapore, 5 Second Hospital Ave., Singapore 168938, Singapore; chen.nah.nah@singhealth.com.sg; 3 Department of Conservative Dentistry, School of Dentistry, Jeonbuk National University, Jeonju 54896, Republic of Korea; endomin@gmail.com

**Keywords:** biodentine, cytotoxicity, dental pulp stem cells, mineralization, mineral trioxide aggregate, silicate cement

## Abstract

The liquid extract method is commonly used to evaluate the cytotoxicity and bioactivity of materials. Although ISO has recommended guidelines for test methods, variations in elution period, and shape of samples can influence the biological outcomes. The aim of this study was to investigate the influence of material form and elution period of Biodentine on dental pulp stem cells (DPSCs)’ proliferation and mineralization. Biodentine (0.2 g) discs or powder were immersed in culture media (10 mL) for 1, 3 or 7 days (D1, D3 and D7). The eluents were filtered and used to treat DPSC. The calcium release profile and pH were determined. Cell proliferation was evaluated by MTS for 3 days, and mineralization and differentiation were assessed by alizarin red S staining (Ca^2+/^ng of DNA) and qRT-PCR (MEPE, DSPP, DMP-1, RUNX2, COL-I and OCN) for 14 days. Statistical analysis was performed with a one or two-way ANOVA and post hoc Tukey’s test (pH, calcium release and proliferation) or Mann–Whitney test (α = 0.05). pH and calcium ion release of powdered eluents were significantly higher than disc eluents. Powdered eluent promoted extensive cell death, while the disc form was cytocompatible. All disc eluents significantly increased the gene expression and mineralization after 14 days compared to the untreated control. D7 induced less mineralization and differentiation compared to D1 and D3. Thus, the materials’ form and elution time are critical aspects to be considered when evaluating the bioactivity of materials, since this binomial can affect positively and negatively the biological outcomes.

## 1. Introduction

The direct application of bioceramics for pulp exposure is a conservative measure for preserving pulp vitality. Calcium hydroxide has historically been used as a pulp-capping agent, owing to its antibacterial properties and the induction of reparative dentin. However, they degrade over time, resulting in poor sealing ability and tunnel defects within the formed dentinal bridges [1]. Hence, the development of biomaterials that can stimulate the dentin–pulp complex regeneration and prevent bacterial leakage is of high interest [2]. As such, tricalcium silicate-based materials have emerged as a viable alternative in the management of vital pulp therapy. Biodentine^®^ (Septodont, St. Maur-des-Fossés, France) is a widely utilized tricalcium silicate in endodontic treatment, known for its advantages, including a shorter setting time and non-discoloration of teeth [3]. The biological effects of Biodentine^®^ have been well documented by numerous in vitro and in vivo studies [4,5,6,7]. It has been shown to induce odontoblastic differentiation and enhance mineralization in human dental pulp stem cells (DPSCs) [5,8,9,10]. Furthermore, it has been demonstrated to stimulate odontoblast-like differentiation and the secretion of TGF-β1, a pivotal growth factor and regulator of tissue repair [5]. These biomaterials can stimulate odontoblast-like differentiation and enhance mineralization potential [10,11,12,13].

To predict and elucidate potential clinical behaviors of bioactive cements, their bioactivity and cytocompatibility are assessed by both in vivo and in vitro models [14]. The latter present distinct advantages of shorter experimental duration, lower costs, and less ethical issues allowing the control of experimental variables [15]. Despite the implementation of standards for testing the biological properties of biomaterials [16], the lack of standardization leading to a variety of the experimental set-ups may result in different reported experimental outcomes [15,17]. The liquid extract method has been extensively used for in vitro testing of biocements. In this method, there are no specifications governing the variables involved in production of the eluents. The material can be shaped as discs [11,18,19] or crushed into powder [6,20,21,22]. Likewise, the elution period varied across studies. While some studies used a day elution period [5,6,8,23], others recommend three [11,22,24,25] or even seven days [26,27,28]. The plethora of possible scenarios can result in different outcomes. Eluents of cements obtained from powder (20 mg/mL, three days) decreased cell proliferation [4,22], while other studies observed no changes in the growth rates when cells were treated with eluent from discs [0.14 mg/mL, four days]. Furthermore, cell proliferation rates exhibited distinct patterns when exposed to different dimensions of the material; for instance, a 4 × 3 mm disc led to decreased cell proliferation on the fifth day [19], whereas a 5 × 3 mm disc resulted in increased cell proliferation on the seventh day [9]. Another study reported increased proliferation when cells were grown directly on the surface of the material [29]. 

Although consensus guidelines (ISO-10993-5) are in place with the aim of standardizing test strategies, a spectrum of variables exist today in the evaluation of a material’s cytocompatibility and bioactivity [14]. The variabilities mentioned above make it difficult to understand and benchmark the biological properties of the materials. This is particularly important in the era of technological advances and the rapid introduction of new dental materials into the market, where clinicians depend heavily on readily accessible in vitro test results before clinical implementation. These materials are instrumental in various clinical procedures, ranging from restoration to regenerative treatment. Moreover, evaluation of dental materials, particularly those in direct contact with vital dental tissues, stand as paramount considerations. Therefore, the development of more specific, standardized, and rigorous testing strategies may enhance the validity of test results. In addition, standardization of variables will serve to enhance reproducibility among different research groups and allow for better cross-referencing of results obtained. Hence, the objective of this study was to evaluate the influence of binomial “material form/elution time” on the chemical differentiation and mineralization potential of the eluted solution. The hypothesis to be tested is that there is no difference in the differentiation of dental pulp stem cells (DPSCs), regardless of the material form and elution time used to obtain the test solutions. 

## 2. Materials and Methods

### 2.1. Material and Elution Preparation

Biodentine (Septodont, St. Maur-des-Fossés, France) was mixed in an amalgamator according to the manufacturer’s instructions and loaded into standardized metal disc molds (10 mm in diameter, 1 mm height). After setting at room temperature (12 min), the samples were incubated for 24 h (37 °C, 95% humidity). Each sample was weighed (0.2 ± 0.02 g) and randomly allocated into disc (D) or powder (P) groups. Powdered samples were prepared by grinding the discs using a mortar and pestle for 4 min each. Particle size analysis was performed in three samples (Malvern Hydro 2000S, Malvern Instruments Ltd., Worcestershire, UK) to confirm the reproducibility of the grinding procedure. Thereafter, one disc or 0.2 g of powder was immersed in 10 mL of ultrapure water (for pH and Ca^2+^ release) or culture media (for cell proliferation and mineralization) and incubated (37 °C, 95% humidity) for different elution periods (1, 3 or 7 days). The specimens were agitated during these elution periods. Importantly, the discs were not transferred to fresh distilled water, but were instead maintained continuously in the same solution throughout the experimental process. After the specified period, eluents were filtered through a micropore filter (0.22 μm. Merck Millipore, Burlington, MA, USA) to ensure cells exposed to the extract are not affected by physical particles, thereby providing a clearer understanding of the biological response to the leachate. The following eluates were used for the various assays reported in this work: day 1 powder (P1), day 1 disc (D1), day 3 disc (D3), and day 7 disc (D7).

### 2.2. pH Measurement of Leachate

Biodentine prepared in discs and powdered forms as previously described above was incubated separately in distilled water under standardized conditions (37 °C, 95% humidity). The pH profile was measured with a pH meter (Orion Star A211, Thermo Scientific, Waltham, MA, USA) over 14 days. 

### 2.3. Calcium Release

The amount of calcium ions leached was evaluated via inductively coupled plasma mass spectrometry (ICP-MS). Briefly, on days 1, 3 and 7, 2 mL of the filtered solution was acidified with 2% nitric acid and 50 µL was added to 4.95 mL of UW in an ICP-MS vial (Autosampler vial, Nanonex Technology, Singapore) after calibration with standard solutions. Thereafter, Ca^2+^ release was determined using an ICP mass spectrometer (Optima 5300DV, Perkin Elmer, Alexandria, VA, USA). Experiments were performed with three independent samples for each material form and elution time. 

### 2.4. Cell Culture and Characterization

The use of human dental pulp stem cells (DPSCs, DPF003, All-cells, Alameda, CA, USA) was approved by the NUS Institutional Biosafety Committee (2014-00762) and Institutional Review Board (NUS 2014/2094). The DPSCs were isolated from the third molar of a single donor and cultured in basal growth media [Dulbecco’s modified Eagle’s medium (DMEM, Invitrogen, Thermo Fisher Scientific, Carlsbad, CA, USA)], supplemented with 10% fetal bovine serum (Invitrogen, Thermo Fisher Scientific, Carlsbad, CA, USA) and 1% penicillin/streptomycin (Invitrogen, Thermo Fisher Scientific, Carlsbad, CA, USA), and characterized with fluorescence-activated cell sorting analysis (CD34, CD73, CD90 and CD105), as previously described by our group [30]. 

### 2.5. Cell Proliferation and Differentiation

DPSCs (2 × 10^3^, passage 5) were seeded into 96-well plates and incubated with basal growth media for 24 h. Thereafter, 100 μL of elution medium from each group, composed of DMEM + 10% FBS with Biodentine leachate, was used to incubate the cells for 3 days. Cell proliferation was assessed using the MTS assay (CellTiter 96 Aqueous One Solution; Promega, USA) in a plate reader (Infinite M200, Tecan, Frankfurt, Germany) at 490 nm. DPSCs cultured in DMEM + 10%FBS without Biodentine were used as controls. 

The mineralization potential of the eluents was evaluated via Alizarin red S staining (Sigma-Aldrich, St. Louis, MO, USA). DPSCs (10 × 10^3^, passage 5) were seeded into 24-well plates and incubated with basal growth media for 24 h. Furthermore, 500 µL of elution medium from each group, composed of DMEM + 10% FBS with Biodentine leachate aged for 1, 3, or 7 days, was used to treat the cells. Subsequently, after 7 and 14 days, mineralized nodules were qualitatively and quantitatively assessed. Briefly, cells were washed with phosphate-buffered saline (Invitrogen, Thermo Fisher Scientific, Carlsbad, CA, USA) and fixed in 4% formaldehyde at room temperature for 30 min. After washing with distilled water, cells were stained with 40 mmol/L of alizarin red in distilled water (pH 4.2 maintained with ammonium hydroxide). The plates were left at room temperature for 30 min, washed with distilled water for four times (5 min each), and imaged with an optical microscope (Olympus IX70, Olympus, Shinjuku, Tokyo, Japan). After imaging, 200 µL 10% acetic acid was added to each well and incubated at room temperature for an additional 30 min. Lastly, 200 µL 10% ammonium hydroxide was added to the solution and incubated for 5 min. The absorbance of 100 µL aliquots of the resulting supernatant was measured at 405 nm using a microplate reader (Infinite M200, Tecan, Frankfurt, Germany). The concentration of calcium was normalized against the DNA content, which was determined using DNAzol (#10503027, Invitrogen, Carlsbad, CA, USA) and measured using a spectrophotometer (NanoDrop ND-1000 Spectrophotometer, Thermo Scientific, Waltham, MA, USA). Controls cells were cultured with DMEM + 10%FBS (control media) or osteogenic media [100 nM dexamethasone (Sigma–Aldrich, St. Louis, MO, USA), 5 mM b-glycerophosphate (Sigma–Aldrich, St. Louis, MO, USA), 50 µg/mL ascorbate phosphate (Sigma–Aldrich, St. Louis, MO, USA), and 10% FBS in DMEM.

The gene expression (Table 1) of matrix extracellular phosphoglycoprotein (MEPE), dentin matrix acidic phosphoprotein 1 (DMP-1), dentin sialophosphoprotein (DSPP), Runt-related transcription factor 2 (RUNX2), collagen I (COL I), and osteocalcin (OCN) was determined by quantitative polymerase chain reaction (qRT-PCR) from cells treated with disc eluents after 7 and 14 days (*n* = 3). Briefly, cells were harvested, and total RNA was isolated (Purelink RNA Mini Kit, Invitrogen, Thermo Fisher Scientific, Carlsbad, CA, USA). Following that, cDNA synthesis was performed (iScript RT Supermix, Bio-Rad, Hercules, CA, USA). The ΔΔCq method was used to calculate the relative gene expression from quantification cycle (Cq) values obtained by quantitative real-time PCR (qRT-PCR) analysis. Three independent qRT-PCR reactions were performed for each sample. 

### 2.6. Statistical Analysis

All the tests were performed in independent triplicates. Statistical analyses were performed with a one or two-way ANOVA and post hoc Tukey’s test (pH, calcium release and proliferation) or Mann–Whitney test (qRT-PCR and calcium nodule deposition). Tests were pre-set at a significant level of 5% (SPSS v. 20.0, New York, NY, USA).

## 3. Results

### 3.1. pH Variation, Calcium Release and Cell Proliferation

**Figure 1:**
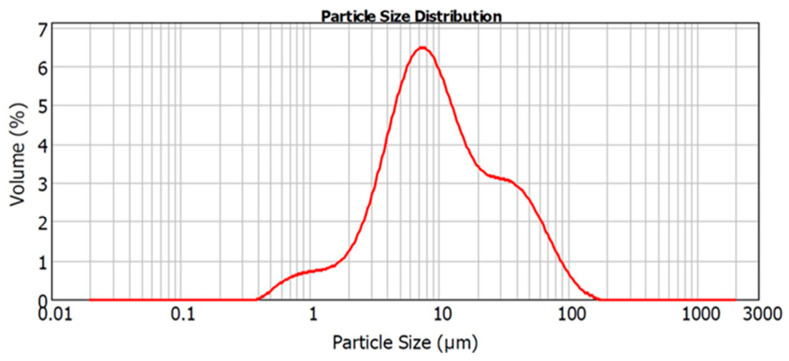
Representative particle size analysis (distribution by volume) of the powdered Biodentine samples after 4 min of grinding by mortar and pestle.

Figure 1 and Table 2 shows the average particle size for the powder particles used in this study. The distribution width of particle sizes within the sample is characterized by D (10), D (50), and D (90) diameters, where the D (90) diameter indicates that 90% of the particles are smaller, and 10% are larger than the specified size. 

**Figure 2:**
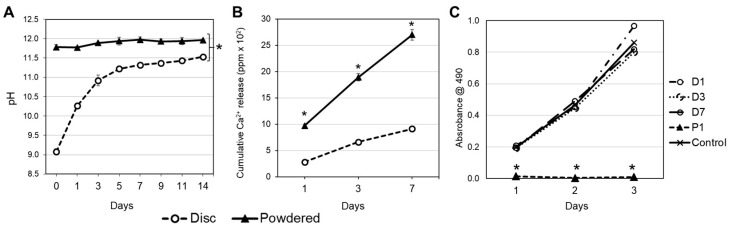
(**A**). Measurement of pH for powder and disc samples in distilled water over 14 days. (**B**). Calcium release. There were significant increases in the amount of calcium ions from the samples from day 1 to day 7 for both disc and powdered forms. (**C**). Proliferation of DPSCs treated with different eluents were assessed for 3 days. The MTS assay showed higher proliferation at day 3 for D1 compared to D3, D7, and control (basal growth medium), which were similar. No DPSC proliferation was observed in the P1 group at all time point (* denotes statistically significant difference of *p* < 0.05 between the groups, two-way ANOVA and Tukey’s test)**.**

Figure 2A shows the variation in pH as a function of material form. The pH of Biodentine in powdered form was significantly higher than that of Biodentine in disc form at all time points studied (*p* < 0.05). For powder form, pH increased marginally from 11.77 ± 0.08 at day 0 to 11.96 ± 0.03 at day 14, but there was no significant increase across all time points studied (*p* > 0.05). For the disc form, pH increased from 9.08 ± 0.07 at day 0 to 11.53 ± 0.02 at day 14; there were significant increases in pH from day 0 to 5 (*p* < 0.05), but no significant increases thereafter. 

Figure 2B illustrates the cumulative release of calcium ions over the elution period for both disc and powdered cements. In our study, the non-cumulative Ca^2+^ ion concentration for the powder group was approximately 970 ± 32.0, 929 ± 22.8, and 812 ± 42.2 ppm, while for the disc group, it was around 283.8 ± 19.6, 384 ± 43.4, and 323 ± 26.0 ppm after 1, 3, and 7 days of total preincubation time, respectively. Notably, ion release from the powdered form exceeded that of the discs at all examined time points (*p* < 0.05). By day 7, the powdered group exhibited a 2.5-fold increase in calcium ion release compared with the disc group. For the disc samples, there was a significant increase in calcium ion release between days 1 and 3 (*p* < 0.05), with no further significant increase thereafter (Figure 2B). Conversely, the powdered forms displayed a significant increase in calcium ion release at all time points (*p* < 0.05).

The effects of the form and eluent duration on DPSC proliferation were evaluated using an MTS assay. Cell proliferation (Figure 2C) increased significantly (*p* < 0.05) over 3 days when cells were treated with eluents obtained from discs (D1, D3 and D7), with no significant difference found among these groups and the control (DMEM only, *p* > 0.05). No proliferation was observed when DPSCs were treated with eluents obtained from the powdered form (P1 in Figure 2C).

### 3.2. Mineralization Potential and Gene Expression

**Figure 3:**
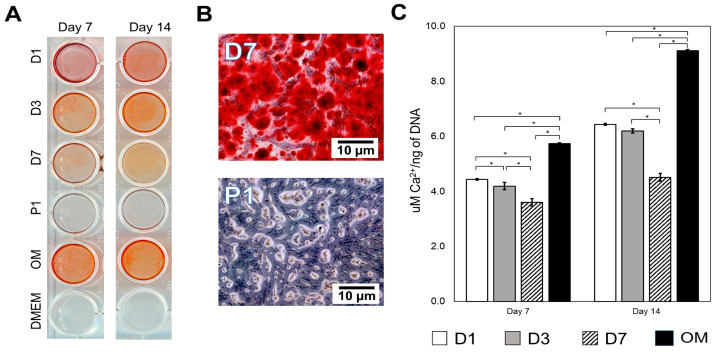
Mineralization potential. (**A**). The macroscope image of cells treated with all eluents obtained from disc group (D1, D3 and D7) after 7 and 14 days. There were no deposits for both powdered group (P1) and basal growth culture medium (DMEM +10%FBS, control). (**B**). Representative microscopic image of DPSCs treated with different eluents after 14 days for P1 and D7 eluent. (**C**). The mineralization potential was quantitatively assessed by alizarin red S staining, which presented lower amounts of Ca per ng of DNA in the eluent from discs compared to osteogenic media. The positive control was osteogenic media (OM), and the negative control was DPSC cultured in basal growth media. (* represents statistical significance between groups, *p* < 0.05)

Mineralization potential was assessed using Alizarin red S staining after treating the cells with eluents from 7 and 14 days. Figure 3A shows that there was no calcium deposition with powdered (P1) and control (DMEM) groups for both 7 and 14 days. On the other hand, the eluents obtained from all disc groups (D1, D3 and D7) presented with the characteristic red stain representing mineralization (Figure 3A). After 14 days, optical microscopy revealed intense calcium nodule deposition for the eluents obtained from all disc’s groups, while only cellular structures with indistinct remained morphology for P1 (Figure 3B). There were significant increases in the amount of calcium ions (Ca^2+^) per ng of DNA from day 7 to 14 for all disc groups analyzed (*p* < 0.05, Figure 3C). 

**Figure 4:**
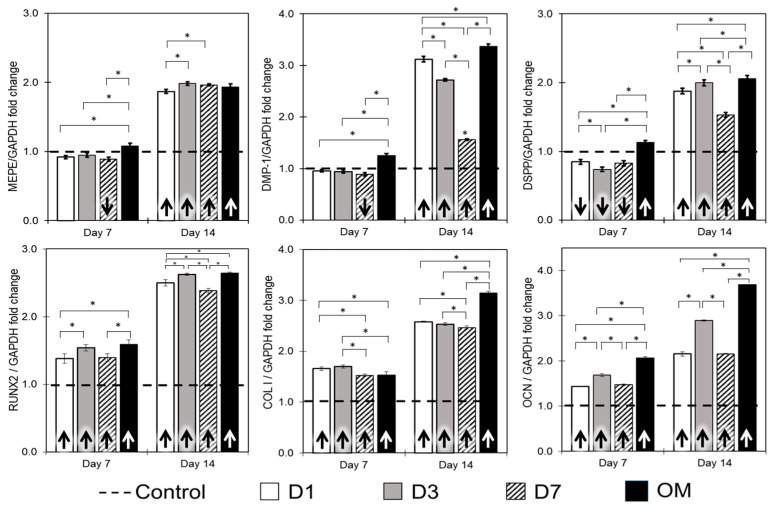
Gene expression. DPSCs were treated with eluents obtained from discs up to 14 days, and the expression of odonto- and osteo-related genes were analyzed using quantitative real-time polymerase chain reaction (qRT-PCR). There was no increase in the expression of all the odontogenic genes tested at 7 days compared to the control (basal growth medium, dashed line). After 14 days, there were significant increases in all genes tested for all the eluents obtained from discs. (* above the linking bar denotes statistically significant differences of *p* < 0.05 between the groups; arrows inside the bar denote difference between the group and control).

Gene expressions for putative markers of odontoblastic (MEPE, DMP-1, DSPP) and osteoblastic differentiation (RUNX2, COL I and OCN) were assessed in cells treated with eluents obtained from discs (Figure 4). There was no increase for all the odontogenic genes tested at 7 days. After 14 days, all the eluents tested promoted higher expression of all odontogenic genes compared to the control (basal growth medium, dashed line). Conversely, all the eluents promoted significant increases in the expression of all osteoblastic-related genes at both time points tested (*p* < 0.05).

## 4. Discussion

Bioactive cements are widely used in pulp therapies (direct pulp capping, indirect pulp capping, pulpotomy), apexification, perforation repair, and others [31,32,33]. However, the literature presents great variability in the experimental set-up used to test the biological behavior of such materials. The eluents used to treat the cells are often obtained by soaking powder and disc-shaped specimens for periods of time that can range from one until seven days [5,6,8,11,20,21,22,23,26,27]. These variabilities often provide different outcomes that compromise the estimation of the bioactive character of the cements [22,29,34]. Here, we tested whether the different sample forms and time used to produce eluents would affect the proliferation, differentiation, and mineralization of DPSCs. 

Bioactive cements can be mixed with various vehicles, such as propylene glycol, distilled water, anesthetic solution, saline solution, chlorhexidine, chitosan, and antibiotics [12,35,36,37]. Distilled water (DW) served as the reference group for comparison due to its well-established efficacy as a vehicle for tricalcium silicate cement and its widespread use by manufacturers [36]. The pH value of Biodentine discs (5 mm × 10 mm) in DW has been reported to range from 11.2 after 1 day to 11.3 after 7 days [36], and other study disc size (4 mm × 5 mm) showed 9.65 to 11.5 after 14 days [38]. A Biodentine powder (5 mg/mL) showed a mean pH value of 11.32 after 7 days [39]. A significant finding in our study was that the pH of Biodentine powder group was markedly higher (11.77 to 11.96) than that of the disc group (9.08 to 11.53) at all time points studied. This higher pH in the Biodentine powder group can be attributed to a substantial release of calcium ions, as evidenced by concentrations of approximately 970 ± 32.0 ppm of Ca^2+^ ions, compared to 283.8 ± 19.6 ppm for the disc group. This calcium ion release, particularly prominent on the first day, contributes significantly to the observed differences in pH between the two groups [40].

Biodentine is a calcium-silicate-based cement that can promote hard tissue regeneration in vivo [7]. In this study, we aimed to investigate the impact of varying elution times and material form on the biological response of DPSCs. DPSCs serve as an appropriate model for testing the application of Biodentine, given its clinical use as a vital pulp capping agent. These cells naturally reside in the vital pulp and can be easily obtained from extracted teeth under local anesthesia, causing minimal damage [41]. Furthermore, DPSCs have the capability to undergo both osteoblastic and odontoblastic differentiation, rendering them an intriguing model for research to test potential of dental cements [10,30]. Herein, we have observed that the eluents obtained with discs were cytocompatible and allowed cell proliferation similar to the untreated control (Figure 1C). Notably, there were no viable cells in the wells treated with the eluent obtained from powdered form for a period as short 1 day (P1). Similarly, for P3 and P7, cell death was observed (data not shown) and was not used for evaluation. The cell death observed in the powdered group (Figure 1C) is consonant with previous observations that powdered eluent from Biodentine (20 mg/mL) was cytotoxic [4,22,25,42]. In fact, the area available for the dissociation of the same mass of material is tremendously high for the powdered specimens compared to the discs. This larger reactive surface of the powder allows for an increased rate of dissociation of Ca^2+^ (Figure 1B). Although Ca^2+^ is an essential regulator of several intracellular processes, the disruption of intracellular Ca^2+^ homeostasis is frequently associated with the onset of irreversible cell injuries. In fact, exaggerated intracellular accumulation of Ca^2+^ can lead to mitochondrial dysfunction, alteration of cytoskeleton organization, and activation of catabolic enzymes [25,43]. Hence, the cell death may be related to both high amounts of Ca^2+^ and high alkalinity of the medium (pH > 11.6 in Figure 1A), as high pH can inhibit enzyme activation in cytoplasmic membranes of DPSCs [8]. Similar observations were made for eluents of mineral trioxide aggregate (MTA) that inhibited the growth and promoted the lysis of murine fibroblasts and macrophages [44]. 

Several bioactive cements (e.g., Ortho-MTA, EndocemZr, MTA and Biodentine) have been shown to induce DPSCs and human odontoblast-like cells to secrete mineralized matrix [10,12,26,45]. Despite these relevant findings, the studies often present different experimental set-ups that make it difficult to unveil the extent of the biological effects promoted by the materials. Unsurprisingly, there was no mineralization in the cells treated with P1, since this form has promoted extensive cell death (Figure 1C and Figure 2B). For the disc form, there were significant differences in the extent of mineralization observed for the elution times studied. Interestingly, cells treated with D7 presented lower mineralization compared to D1 and D3 for both time points evaluated (Figure 3A,C). These results may be related to the significantly higher pH and calcium release observed for D7 (Figure 1A,B). Although this seems to be counterintuitive, it is possible that the high alkalinity of the D7 media has surpassed the optimal alkaline environment that can upregulate alkaline phosphatase activity and enhance mineralization [46]. Moreover, extreme changes in extracellular osmolality may alter normal cell function [47]. Besides, the higher availability of Ca^2+^ in D7 (Figure 1B) may have negatively influenced the homeostasis of the cells. Indeed, high concentrations of hydroxyl ions may suppress cell activity and arrest vital processes, as observed in pulp tissue in direct contact with calcium hydroxide [48]. Additionally, the longer elution time (D7) did not translate into increased expression of DMP-1 and DSPP (Figure 3), which are putative markers for odontoblastic differentiation [10]. This is a significant finding, since the elution times can be purposely adjusted to favor or compromise the biological performance of materials depending on their calcium content.

Our results showed that all the eluents failed to increase the expression of odontogenic-related genes (MEPE, DSPP and DMP-1) after 7 days compared to the untreated control. Nonetheless, there were significant increases in the expression of these genes after 14 days (Figure 3). Similar increases have been observed for DSPP and DMP-1 gene expression in human dental pulp cells cultured directly on the surface of BioAggregate and MTA for 7 days or after being treated with Biodentine (10 mg/mL, discs eluted for 7 days) for two days [49]. Eluent from Biodentine (1 mg/mL, disc eluted for 1 h) also increased the gene expression of DMP-1 in stem cells from human deciduous teeth after 21 days [50]. Herein, there were significant increases for RUNX2, COL I and OCN compared to the untreated control for both time points evaluated (Figure 3). The early expression of osteogenic-related markers seems to be consistent across studies when pulp cells are treated with calcium-silicate-based cements. Previously, high gene expression of OCN was observed in human dental pulp cells exposed to BioAggregate, iRoot BP Plus and MTA for 7 days [49], and positive expression of osteopontin protein was observed at an early timepoint in pulps of dogs treated with MTA and Biodentine [51]. The differences in the expression profiles may be explained by the fact that OCN and OPN markers are expressed in late polarizing odontoblasts and secretory odontoblasts, while DSPP and DMP-1 expression is higher in terminally differentiated and secretory or functional odontoblasts [52,53,54]. However, it is essential to recognize that the in vitro conditions discussed above significantly contrast with the dynamic pH variations encountered in clinical settings. In dental scenarios, pulpal and periapical inflammation often lead to a notable reduction in pH, creating an acidic environment [55]. As a result, bioceramic materials such as Biodentine are frequently exposed to inflamed tissues with acidic characteristics during various clinical procedures. It is worth noting that a study involving Biodentine application within the lumen of bovine dentine discs revealed interesting findings when stored in different environments, either saline or citric acid (pH 5.4), for 24 h. The results indicated a higher cell density and a greater number of adherent cells on dentin discs stored in an acidic environment than those stored in saline or no specific storage medium [29]. This highlights the intricate interplay between material behavior and environmental factors, emphasizing the need for a comprehensive understanding of these dynamics for clinical applications [56].

Despite the implementation of ISO consensus standards, there remains room for variability between in vitro test methods [16]. Different material forms and elution times may produce different results for the same material tested. As shown, the liquid extract method, which is widely used for assessing biological responses to materials, lacks specific ISO 10993-5 guidelines regarding the form of the test material. Researchers have tested various material forms, either allowing samples to set as discs and cylinders, or crushing them into powdered extracts. Studies show contrasting results for different forms of the same material, indicating varied cell responses. However, there is a notable absence of research comparing results between different material forms, highlighting the need for standardized approaches in biocompatibility testing under ISO guidelines. Our results demonstrated that eluents obtained with Biodentine discs exhibited cytocompatibility, fostering cell proliferation comparable to the untreated control (Figure 2C). Importantly, the powdered form, even with a short elution period of 1 day, showed no viable cells in the treated wells.

The elution period, or extraction time, in biocompatibility testing involves incubating materials in culture media, while ISO 10993-5 outlines standard conditions but allows variations with justification [16]. Studies vary in elution period from 1 day to 1 week, impacting cell responses. For instance, a 1-week incubation of dental materials showed similar viability [26], while 24 h exposure to tricalcium silicate powder enhanced cell proliferation [57]. Furthermore, our study demonstrated that different elution times resulted in variations not only in cell viability, but also in mineralization and odontogenic gene expression. These findings emphasize the multifaceted impact of elution time on the biological responses assessed in biocompatibility studies, reinforcing the need for careful consideration of this factor in the experimental design and interpretation of results.

## 5. Conclusions

Within the limitations of this study, eluents obtained from Biodentine-induced DPSCs secrete calcium nodules and increase the expression of several genes associated with mineral-secreting cells. However, the observed disparities in outcomes based on the material form (disc or powder) and elution timeframes used in the generation of eluents have led to the rejection of the initial study hypothesis. These results highlight a pressing concern regarding the lack of standardization in the methodologies employed for assessing the bioactivity of calcium silicate-based cements. Although we acknowledge the deliberate unspecific nature of ISO 10993-5, the unreliability of test results is unacceptable, and poses potential issues for patients, users, and manufacturers. To establish reliable and reproducible means for comparing and testing dental cements, it is essential to carefully revise standards and urge laboratory/manufacturers to adhere strictly to them. This is crucial to ensure equitable comparisons among materials, and to mitigate the risk of either underestimating or overestimating their biological effects.

## Figures and Tables

**Table 1:** Oligonucleotide primer sequences used in the RT-PCR.

Primer	Sequence
MEPE Forward	GGCCAGTGACTGCGATTAAAC
MEPE Reverse	CCTTCGAGTGTGCTTTAGCAT
DMP-1 Forward	CTCCGAGTTGGACGATGAGG
DMP-1 Reverse	TCATGCCTGCACTGTTCATTC
DSPP Forward	TGGCGATGCAGGTCACAAT
DSPP Reverse	CCATTCCCACTAGGACTCCCA
RUNX2 Forward	CACTGGCGCTGCAACAAGA
RUNX2 Reverse	CATTCCGGAGCTCAGCAGAATAA
COL I Forward	CTGACCTTCCTGCGCCTGATGTCC
COL I Reverse	GTCTGGGGCACCAACGTCCAAGGG
OCN Forward	ATGAGAGCCCTCAGACTCCTC
OCN Reverse	CGGGCCGTAGAAGCGCCGATA

**Table 2:** Distribution of powder particle sizes (diameter in µm).

D10	D50	D90
2.79 ± 0.04	9.73 ± 0.95	46.82 ± 6.71

## Data Availability

The data are available in the article.
